# Hospital Expenditure.—The Commissariat

**Published:** 1896-08-08

**Authors:** 


### THE INSTITUTIONAL WORKSHOP.

### HOSPITAL EXPENDITURE.—THE COMMISSARIAT.

### XXII.—THE BREWING OF BEEF TEA.

Before completing the experiments for testing the comparative beef tea value of English and foreign meat, it was thought desirable to ascertain what proportion of water added to the meat gave the best results. With this view four more samples of tea were brewed as follows :—

| | | |
|---|---|---|
| G | ½ pint water to 1lb. Scotch beef | Selected from several lbs. of finely minced meat. |
| H | ½ " " " American " | |
| J | 1 " " " Scotch " | |
| K | 1 " " " American " | |

The method of brewing was identical with that employed for samples C and D, viz., infusion for an hour in cold water and then simmering in closed jars in the oven for six hours. Though great care was taken to avoid loss by evaporation, it was found on measuring the liquids that they had decreased to the following unequal extent :—

| | | |
|---|---|---|
| G | lost 149 c.c. out of an original volume of | 284 c.c. or ½ pint. |
| H | " 93 c.c. " " " | 284 c.c. or ½ " |
| J | " 66 c.c. " " " | 568 c.c. or 1 " |
| K | " 145 c.c. " " " | 568 c.c. or 1 " |

As this loss was not made up before analysis, it must be taken into consideration in studying the following report, which would otherwise appear to present somewhat contradictory results. That all the teas had decreased in volume during the cooking process instead of increasing, as in the former experiments, is due to the fact that small quantities only were in this instance prepared, and that the amount of evaporation was therefore more than sufficient to counterbalance the gain from the juices of the meat. It is obvious that those brews which lost most by evaporation will present measure for measure a higher percentage of extractives than the others. Hence we find in the first case, or double strength tea, the Scotch standing first, and in the second or single strength tea, the American superior.

REPORT SHOWING THE QUANTITIES PRESENT IN 100 C.C. OF THE TEAS AS RECEIVED :—

| | G Scotch. (Double Strength.) | H Amer. (Double Strength.) | J Scotch. (Single Strength.) | K Amer. (Single Strength.) |
|---|---|---|---|---|
| | Grams per 100 c.c. | | | |
| Original volume in c.c. | 135 | 191 | 502 | 423 |
| Suspended matter ... | 4·343 | 4·016 | 6·069 | 5·296 |
| Total extract ... ... | 7·454 | 5·102 | 3·394 | 3·640 |
| Mineral matter ... | 1·052 | 0·990 | 0·630 | 0·618 |
| Total nitrogen .. ... | 0·836 | 0·596 | 0·384 | 0·415 |
| Albumen ... ... | 0·120 | 0·0824 | 0·0208 | 0·0288 |
| Albumose ... ... | 3·182 | 2·14 | (?) | 1·965 |
| Nitrogen soluble in water ... ... ... | 0·647 | 0·320 | 0·203 | 0·237 |
| Nitrogen in phosphotungstic ... ... | 0·647 | 0·3802 | 0·1058 | 0·216 |

The amount of meat used being in all four cases the same, the comparative strength of the teas must be measured by the proportion of their volumes after evaporation. G ought, according to this, to contain between three and four times more food properties than J, and H rather more than double those of K, if the extra proportion of meat to water is to be considered justified by results. But this is the case in two tests only out of the whole number, and those dealing with very minute quantities.

It will be interesting to compare these figures with an analysis of beef tea made by Dr. Phillips Bedson, Professor of Chemistry in the Durham College of Science at Newcastle-on-Tyne, an account of which appeared in THE HOSPITAL for July, 1895. Dr. Bedson first made his beef tea in what he calls "the ordinary way," viz., "allowing very nearly a pound to a pint; the beef was allowed to soak in the water for some hours, then both together were gradually warmed to near boiling point." The result (omitting fat) was :—

| | Grams per c.c. | Grains per pt. |
|---|---|---|
| Albumen ... ... ... ... ... | ·499 | 43·7 |
| Gelatine, &c. (insoluble in alcohol)... | ·894 | 78·29 |
| Extractives (soluble in alcohol) ... | ·862 | 75·7 |
| Salts ... ... ... ... ... | ·361 | 31·6 |
| Total solids or extract ... ... | 2·628 | 229·29 |

The results of an entirely cold extraction made with ten ounces of beef to one pint were distinctly unsatisfactory, the result working out as follows :—

| | Grams per c.c. | Grains per pt. |
|---|---|---|
| Albumen ... ... ... ... ... | ·449 | 39·3 |
| Gelatine, &c. (insoluble in alcohol)... | ·243 | 21·3 |
| Extractives (soluble in alcohol) ... | ·737 | 64·4 |
| Salts ... ... ... ... ... | ·076 | 6·6 |
| Total solids or extract ... ... | 1·505 | 131·6 |

Dr. Bedson's next trial was in the proportion of a pound to one pint. A pound of finely minced beef was first mixed with half a pint of cold water and left for twelve hours. The liquid was then strained off and cooked, and a second half-pint of water added. This was allowed to remain with the meat for three hours, and then the whole, meat as well as liquor, gradually heated to near the boiling point. This tea was then strained off, and added to the first with sufficient water to allow for any loss by evaporation. The analysis of this showed :—

Proportions one pound to a pint.

| | Grams per c.c. | Grains per pt. |
|---|---|---|
| Albumen ... ... ... ... ... | ·449 | 67 8 |
| Gelatine, &c. (insoluble in water) | ·386 | 33·78 |
| Extractives (soluble in alcohol) ... | 1·153 | 100·9 |
| Salts ... ... ... ... ... | ·4009 | 35·08 |
| Total extract or solids, omitting fat ... ... ... ... ... | 2·3889 | 237·56 |

It is not possible to compare these results in full with those of Dr. Rideal, since the minute distinctions of chemical properties shown in the analyses of the latter cannot be summarised to correspond with Dr. Bedson's table. It will be interesting, however, to contrast the proportion of total extract or solids and of mineral matter or salts yielded by the beef teas made in so dissimilar a manner. The total extr ct n both cases represents the total soluble matter obtaine from the filtered tea by evaporating off all the wat r i contains —in popular language, the "goodness" of the tea.

| | Dr. Bedson's Slow Infusion. | Dr. Rideal's Scotch J. | Dr. Rideal's American K. |
|---|---|---|---|
| Total extract grams per c.c. | 2·628 | 3·394 | 3·640 |
| Total extract grains per pint ... ... ... | 229·29 | 296·975 | 318·5 |
| Mineral matter or salt, grams per c.c. ... ... | 0·361 | 0·63 | 0·618 |
| Mineral matter or salt, grains per pint ... ... | 31·6 | 55·125 | 54·075 |

Making all due allowance for the evaporation which tended to increase the comparative strength of Dr. Rideal's infusions, it will be seen that what may be called the old-fashioned method of brewing in jars in the oven has a distinct advantage over the lengthy process of nearly cold infusion described above. (1) A second brew made by infusing the strained beef in the same jars with sufficient hot water to make up for the losses by evaporation would, if added to the first, have made up the full value of Dr. Rideal's teas and rendered the results even more satisfactory.

Returning to the four samples of beef tea already examined, we have the opportunity of comparing the amounts extracted from one pound of beef, foreign and English respectively, by (*a*) half a pint of water and (*b*) one pint. As all the water is evaporated before analysis, the amount of evaporation during cooking does not here come into question.

| | G. | H. | J. | K. |
|---|---|---|---|---|
| Total suspended matter | 4·343 | 4·016 | 6·069 | 5·296 |
| Total extract | 10·03 | 9·744 | 17·037 | 15·395 |
| Mineral matter | 1·42 | 1·89 | 3·16 | 2 61 |
| Total nitrogen | 1·128 | 1·137 | 1·926 | 1·756 |
| Albumen | 0·162 | 0·158 | 0·104 | 0·122 |
| Albumose | 4 296 | 4·087 | (?) | 8 312 |
| Peptone | 0 837 | 0·568 | — | — |
| Total albuminoids | 5·465 | 4·538 | 3·319 | 5·712 |
| Nitrogen in albuminoids | 0·874 | 0 726 | 0·531 | 0·914 |
| Nitrogen as flesh bases | 0·4445 | 0 6118 | 1·0199 | 1·005 |

Dr. Rideal's concluding remarks will be read with great interest as an important contribution to a vexed question, which by his repeated analyses he has done much to raise out of the region of controversy.

"A comparison of the two sets of figures does not enable one to draw any conclusion as to a difference existing between the two kinds of meat employed in their manufacture, and I am therefore of opinion that beef teas of similar quality can be made from meat of any source."

With respect to double and single strength beef tea his opinion is as follows:—

"When a large quantity of water relatively to the meat is employed the extraction or solution of both the mineral and organic constituents of the meat are more complete, and therefore more of the meat extractives are obtained when the ordinary method of making beef tea from meat in the ratio of one pound of meat to one pint of water than when double the strength is prepared."

No experiments were made on the relative value of beef tea made with three-quarters of a pound of meat to one pint, but the evidence shows that made in two infusions, first in the proportion of three-quarters of a pint to three-quarters of a pound, and then second, the remaining quarter of a pint of water added hot to the meat for a second brew in the same vessel after the liquid had been strained off, every particle of food value would be extracted, and the two brews together would quite equal in strength tea made from one pound to one pint, without a second infusion.

[1] Excellent beef tea was, in fact, made from the remains, but was not subjected to analysis.

